# Spred2-deficiency enhances the proliferation of lung epithelial cells and alleviates pulmonary fibrosis induced by bleomycin

**DOI:** 10.1038/s41598-020-73752-3

**Published:** 2020-10-05

**Authors:** Akina Kawara, Ryo Mizuta, Masayoshi Fujisawa, Toshihiro Ito, Chunning Li, Kaoru Nakamura, Cuiming Sun, Masaki Kuwabara, Masahiro Kitabatake, Teizo Yoshimura, Akihiro Matsukawa

**Affiliations:** 1 Department of Pathology and Experimental Medicine, Graduated School of Medicine, Dentistry and Pharmaceutical Science, Okayama University, 251 Shikata, Kita-ku, Okayama, 700-8558 Japan; 2 Department of Immunology, Nara Medical University, Kashihara, 634-8521 Japan

**Keywords:** Mechanisms of disease, Cell signalling

## Abstract

The mitogen-activated protein kinase (MAPK) pathways are involved in many cellular processes, including the development of fibrosis. Here, we examined the role of Sprouty-related EVH-1-domain-containing protein (Spred) 2, a negative regulator of the MAPK-ERK pathway, in the development of bleomycin (BLM)-induced pulmonary fibrosis (PF). Compared to WT mice, Spred2^−/−^ mice developed milder PF with increased proliferation of bronchial epithelial cells. Spred2^−/−^ lung epithelial cells or MLE-12 cells treated with spred2 siRNA proliferated faster than control cells in vitro. Spred2^−/−^ and WT macrophages produced similar levels of TNFα and MCP-1 in response to BLM or lipopolysaccharide and myeloid cell-specific deletion of Spred2 in mice had no effect. Spred2^−/−^ fibroblasts proliferated faster and produced similar levels of MCP-1 compared to WT fibroblasts. Spred2 mRNA was almost exclusively detected in bronchial epithelial cells of naïve WT mice and it accumulated in approximately 50% of cells with a characteristic of Clara cells, 14 days after BLM treatment. These results suggest that Spred2 is involved in the regulation of tissue repair after BLM-induced lung injury and increased proliferation of lung bronchial cells in Spred2^−/−^ mice may contribute to faster tissue repair. Thus, Spred2 may present a new therapeutic target for the treatment of PF.

## Introduction

Idiopathic pulmonary fibrosis (IPF) is one of the lung interstitial pneumonias, defined as interstitial fibrosis that grows chronically and progressively^1–3^. It is generally thought that IPF is a consequence of multiple events involving genetic and environmental risk factors, and repetitive microinjuries to ageing alveolar epithelium result in activation of alveolar epithelial cells to secrete fibrogenic growth factors, cytokines, and coagulants, leading to the recruitment and activation of myofibroblasts. These myofibroblasts deposit increased amounts and altered extracellular matrix, with increased biomechanical stiffness, further contributing to myofibroblast activation in a positive feedback loop. Since patients with IPF often develop dyspnea, it heavily damages their daily life activities^1–3^.

Histologically, IPF is characterized by the remodeling of the alveolar architecture. Notably normal alveolar epithelial cells and endothelial cells are lost and replaced by fibroblasts, with an appearance of usual interstitial pneumonia (UIP) pattern^1,3^. The causes of IPF are mostly unknown except for cases, such as drug-induced PF; therefore, effective therapies for IPF are lacking and patients are treated just to reduce their symptoms^4^. To establish new therapies, a number of studies have been conducted using the murine bleomycin (BLM)-induced PF model, a commonly used model of human IPF. Bleomycin (BLM) is a chemotherapeutic antibiotic, produced by the bacterium “Streptomyces verticillus”^5,6^. Its primary clinical use is as an antitumor antibiotic for various carcinomas and lymphomas. BLM-induced toxicity occurs predominantly in the organs, such as the lung, skin, and mucous membranes, due to the lack of the BLM-inactivating enzyme, bleomycin hydrolase in these tissues. Pulmonary fibrosis is a well-known side effect of BLM, because the lung expresses very low levels of this enzyme. Instillation of BLM to the lung of animals causes pulmonary injury, inflammation, and subsequent fibrosis^7,8^.

Mitogen activated protein kinases (MAPKs), including extracellular signal-regulated kinase (ERK), c-jun N-terminal kinase (JNK), and p38 kinase (p38 MAPK), regulate important cellular processes, including gene expression, cell proliferation, cell survival and death, and cell motility^9^. In general, p38 MAPK and JNK are activated by environmental stresses and inflammatory cytokines and play important roles in cytokine expression and apoptosis, whereas ERK is activated by mitogenic stimuli and stimulates DNA synthesis and cell proliferation and protects cells from apoptosis^10^. ERK, JNK, and p38 MAPK were all activated in lung tissues from patients with IPF compared with normal lung parenchyma^11^. Inhibition of ERK with the MEK inhibitor PD98059 reduced the lung injury and inflammation after the intratracheal BLM administration in mice. Inhibition of ERK and p38 MAPK with PD98059 and SB203580, respectively, remarkably reduced the amiodarone-induced proliferative response of human embryonal lung fibroblasts and greatly attenuated α-SMA, vimentin and collagen I protein production^12^. These previous observations suggested an important role of MAPKs in the development of PF, including IPF.

Sprouty-related EVH1 (enabled/vasodilator-stimulated phosphoprotein homology 1)-domain-containing proteins (Spreds) can inhibit Raf activation, resulting in ERK1 inactivation^13,14^. Spred family proteins, including Spred1, Spred2, and Spred3, inhibit the MAPK cascade mediated by fibroblast growth factor receptor and epidermal growth factor receptor, by binding to Ras and consequently inhibiting the phosphorylation of Raf^15^. Particularly, Spred2 is expressed in various tissues, including the lung^16–18^. Our previous studies focusing on Spred2 demonstrated that Spred2 controls the development of lung inflammatory responses by negatively regulating the ERK pathway using an acute lung injury (ALI) model induced by lipopolysaccharide (LPS)^19,20^, leading us to the hypothesis that Spred2-deficiency may aggravates the development of PF.

In the present study, we examined the role of Spred2 in BLM-induced PF and demonstrate, for the first time, that contrary to our hypothesis Spred2-deficiency abates the development of BLM-induced PF with increased proliferation of epithelial cells which may facilitates the repair of injured epithelial cells and reduce subsequent fibrosis.

## Results

### Spred2-deficiency alleviates BLM-induced PF

**Figure 1:**
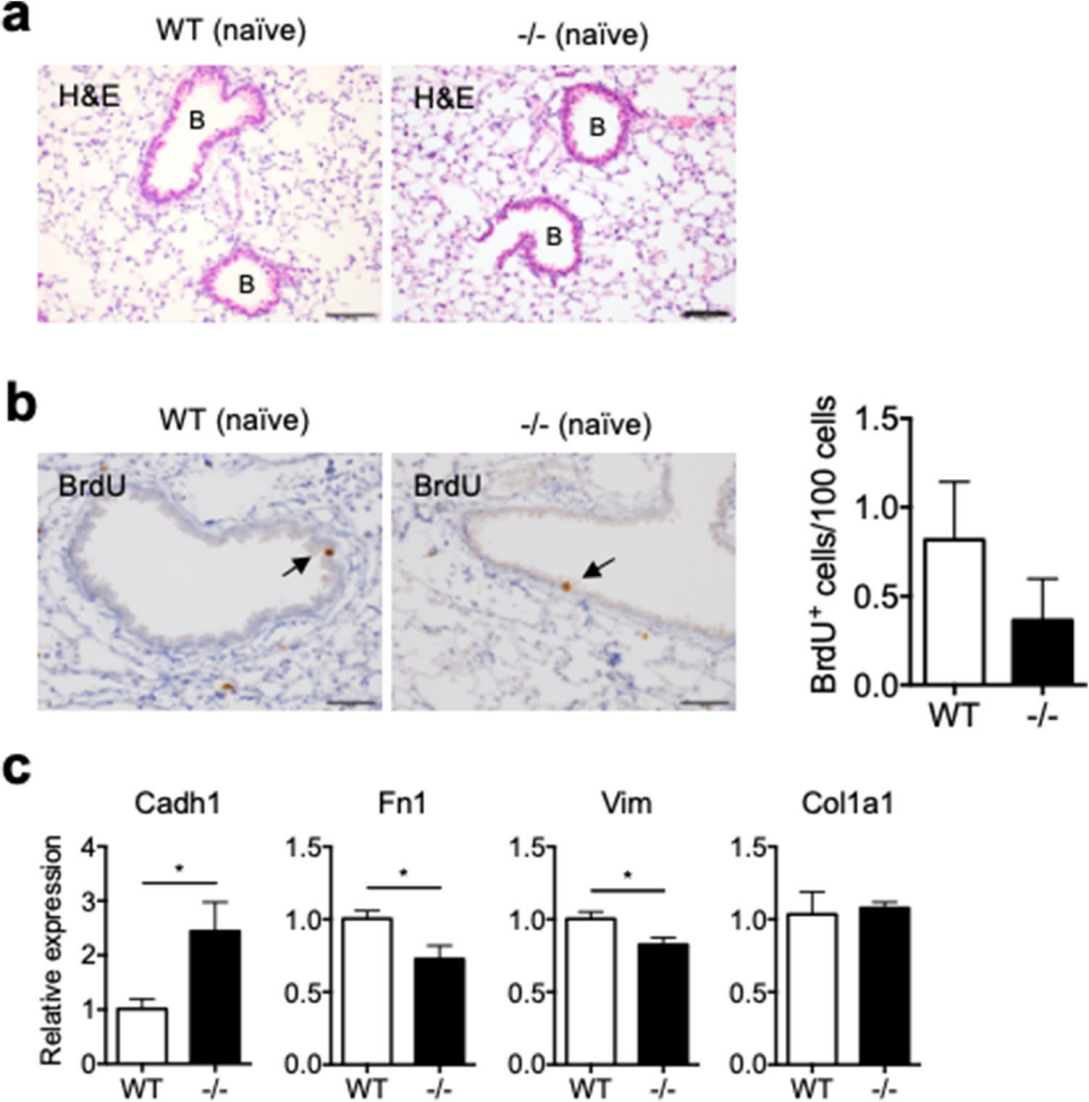
Characterization of lungs from naïve WT and Spred2^−/−^ mice. (**a**) Lung tissues were removed from WT and Spred2^−/−^ mice and examined by H&E staining. Bronchi are indicated by (B). The original magnification was 200×. The scale bars are 100 μm. (**b**) Naïve WT and Spred2^−/−^ mice were intraperitoneally injected with BrdU. Two hours later, mice were euthanized and the incorporation of BrdU was examined by IHC. The number of BrdU^+^ cells in five bronchi were counted and presented as BrdU^+^ cells/100 cells. The results are presented as mean ± SEM. n = 5. The original magnification was 400×. The scale bars are 50 μm. (**c**) The expression of the *Cadh1*, *Fn1*, *Vim* and *Col1a1* gene was examined by qRT-PCR. The results are presented as mean ± SD. n = 4. *p < 0.05. ***p < 0.0001.

We first examined the lung of naïve WT and Spred2^−/−^ mice (Fig. 1a). Histological examination of lung tissue sections showed that there were no detectable differences in the structure or the condition of the lung. Incorporation of BrdU was detected by immunohistochemistry (IHC) in a small number of bronchial epithelial cells, including Keratin 5 (Ker5)^+^ basal cells and Clara cell 10 kD protein (CC10, also known as Secretoglobin 1a1)^+^ Clara cells, in the lung of both WT and Spred2^−/−^ mice (Fig. 1b, Supplementary Fig. S1). We also examined the expression of genes expressed by lung epithelial cells or mesenchymal cells by qRT-PCR. The expression of *Cadherin (Cadh) 1* was slightly increased, whereas the expression of *Fibronectin 1 (Fn1)* and *Vimentin (Vim)* mRNA was slightly decreased. The expression of *Collagen1a (Col1a1)* mRNA was similar (Fig. 1c). Thus, Spred2-deficicncy has little effects on the basal structure or condition of the lung although minor differences in the expression of some genes were found in the lung of Spred2^−/−^ mice.

**Figure 2:**
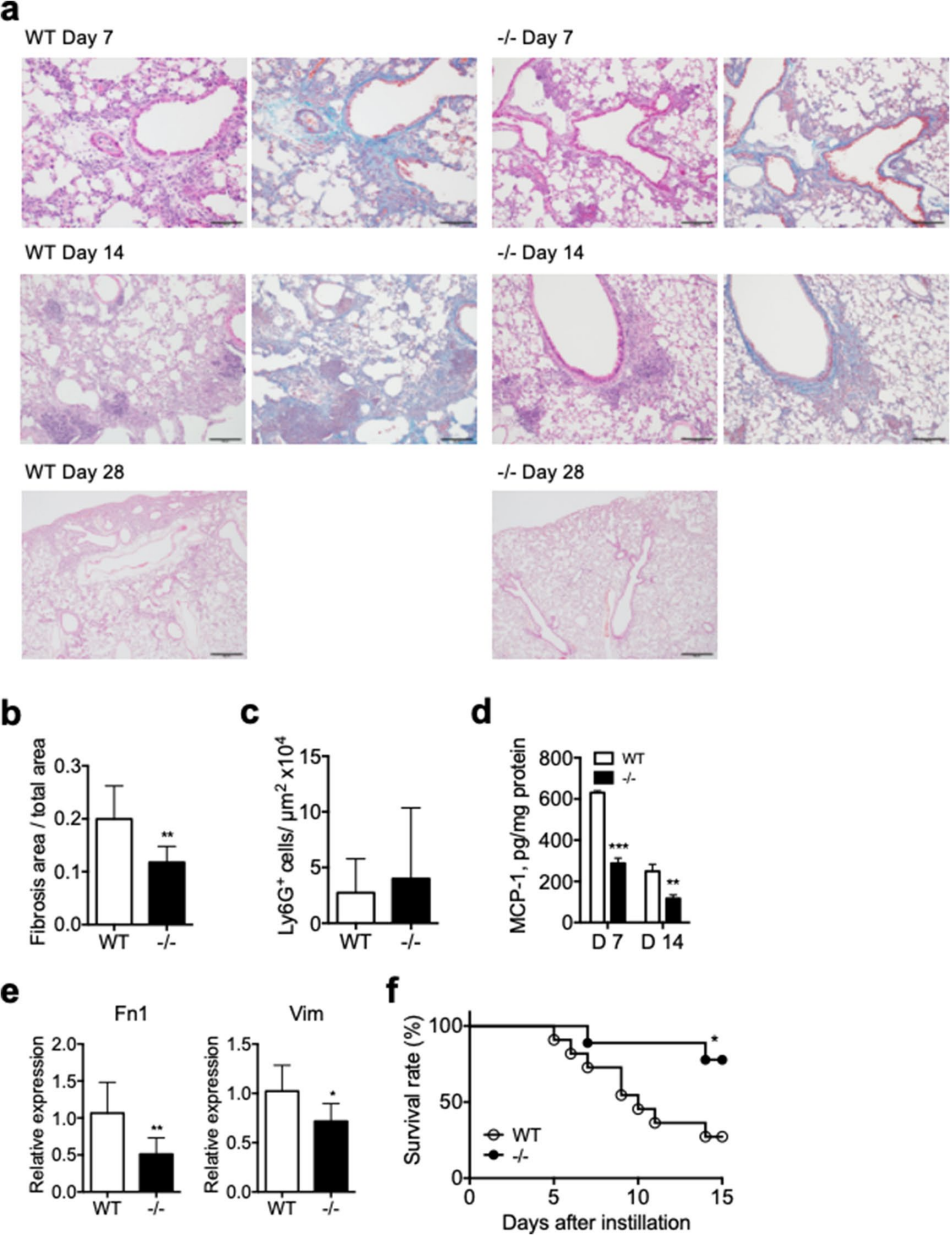
Characterization of lungs from naïve WT and Spred2^−/−^ mice after intratracheal instillation of BLM. Mice were injected intratracheally with 1.5 mg/kg BLM (equivalent to 1.5 U/kg). (**a**) Representative images of lung Sects. 7, 14 and 28 days after BLM administration are shown. H&E staining and Masson’s trichrome staining were performed. The original magnification was 200 × for Day 7 and 14, and 40 × for Day 28. The scale bars are 100 μm for Day 7 and 14, and 500 μm for Day 28. (**b**) The ratios of fibrosis area to total lung area were calculated as described in the M&M section. The results are presented as mean ± SD. n = 5 for WT and 8 for Spred2^−/−^ mice. (**c**) MCP-1 protein levels in the whole lung tissue 14 days after BLM injection were measured by ELISA. The results are presented as mean ± SD. n = 5 for WT and 8 for Spred2^−/−^ mice. *p < 0.05 and **p < 0.01. (**d**) The expression of the Fn1 and Vim gene in the lung 14 days after BLM injection was examined by qRT-PCR. The results are presented as mean ± SD. n = 5 for WT and 9 for Spred2^−/−^ mice. *p < 0.05 and **p < 0.01. (**e**) Mice were injected with 3 mg/kg (equivalent to 3 U/kg) BLT and the survival of mice was examined. n = 11 for WT and 9 for Spred2^−/−^ mice. *p < 0.05.

To examine a potential role of Spred2 in host responses to tissue injury in the lung, we intratracheally administered 1.5 mg/kg BLM in WT and Spred2^−/−^ mice and compared the development of PF. As shown in Fig. 2a, a significant level of PF with alveolar destruction, leukocyte recruitment and collagen fiber deposition (stained blue by Masson’s trichrome staining) was detected in the lung of WT mice by day 7. The level of PF peaked on day 14 with increased collagen fiber deposition but PF was largely resolved with only small fibrotic lesions by day 28. In contrast to WT mice, the deposition of collagen fibers was milder in Spred2^−/−^ mice on day 7 and 14 (Fig. 2a,b) and PF was almost completely resolved by day 28. As far as leukocyte infiltration, a similar level of neutrophil infiltration was detected in the lung of both WT and Spred2^−/−^ mice on day 7 (Fig. 2c). The levels of MCP-1 in the lung of Spred2^−/−^ mice were significantly lower than in WT mice on day 7 and 14 (Fig. 2d). The expression of *Fn1* and *Vim* mRNA was also lower in the lung of Spred2^−/−^ mice on day 14 (Fig. 2e). When the dose of BLM was increased to 3 mg/kg, 8 of 11 WT mice (72.7%) died by day 14 with high levels of PF, whereas only 2 of 9 Spred2^−/−^ mice (22.2%) died (Fig. 2f). These results indicated that Spred2-deficiency alleviates the development of BLM-induced PF with reduced levels of MCP-1 production and collagen fiber deposition.

### Spred2-deficiency increases the proliferation of bronchial epithelial cells in BLM-treated Spred2^−/−^ mice

**Figure 3:**
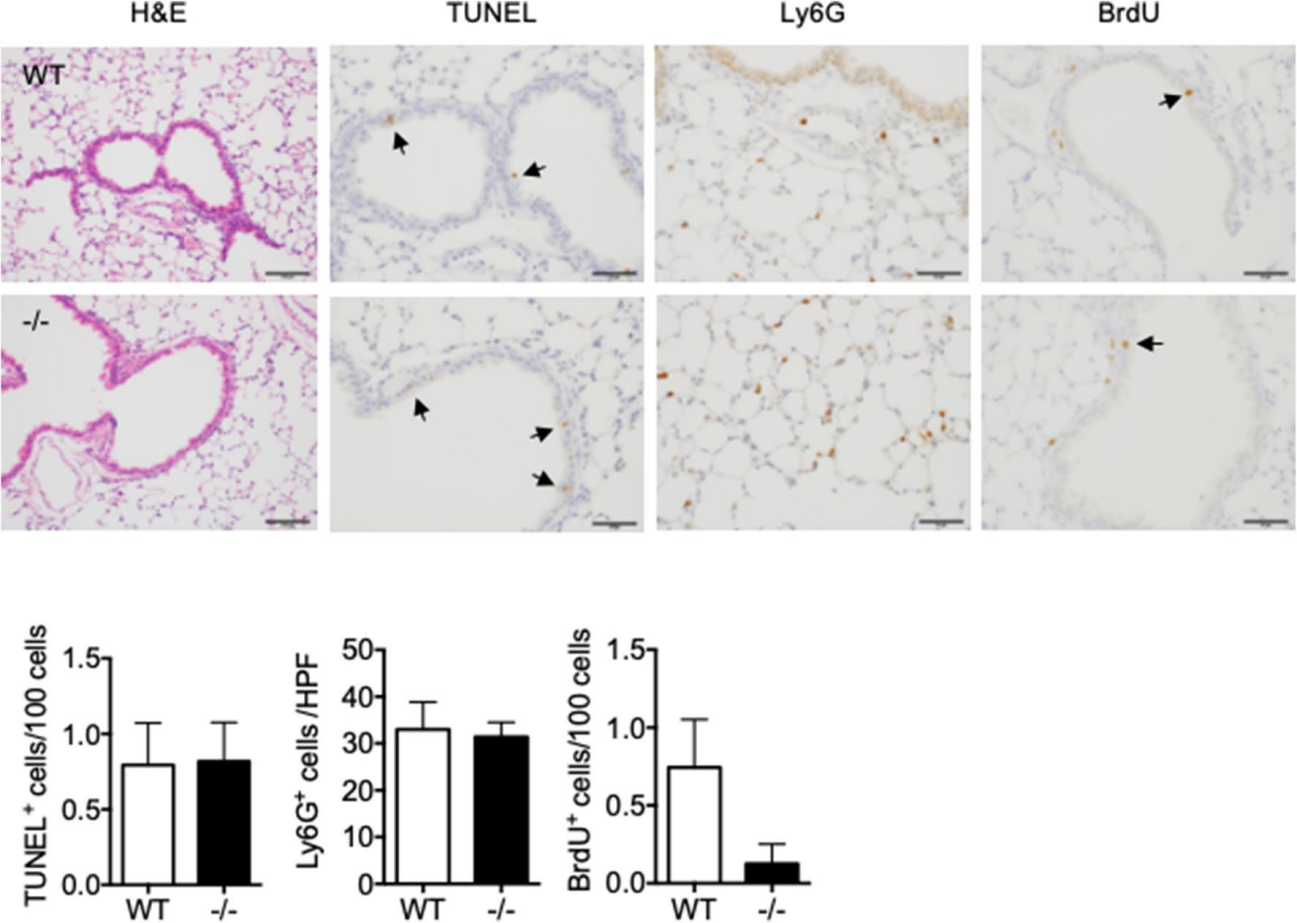
Characterization of lungs from naïve WT and Spred2^−/−^ mice 1 day after BLM administration. Mice were injected intratracheally with 1.5 mg/kg BLM and lung tissues were harvested after 24 h. Tissue sections were subjected to H&E staining, TUNEL and IHC with anti-Ly6G Ab. To detect the proliferation of cells, BrdU was intraperitoneally injected 2 h before the harvest and the incorporation of BrdU was evaluated by IHC. Representative images are shown. The original magnification was 200 × for H&E staining and 400 × for TUNEL, Ly6G or BrdU staining. Arrows indicate positive cells. The number of TUNEL^+^ or BrdU^+^ cells in five bronchi were counted and presented as TUNEL^+^ or BrdU^+^ cells/100 cells. Ly6G^+^ cells in five high power field (400×) were counted and the results are presented as mean ± SEM. n = 5. Scale bars are 100 μm for H&E and Ly6G staining and 50 μm for TUNEL and BrdU staining.

Previous studies demonstrated that both epithelial cells and fibroblasts contribute to the development of PF, and the results of genetic studies suggested that the dysfunction of epithelial cells play a role in the development of lung fibrosis^2^. To elucidate a potential mechanism by which Spred2-deficiency alleviates BLM-induced PF, we first focused on epithelial cells. One day after BLM instillation, similar numbers of apoptotic epithelial cells were detected in both WT and Spred2^−/−^ lungs as shown by TUNEL (Fig. 3). Similar numbers of Ly6G^+^ neutrophils were detected mainly in the alveolar regions at this stage (Fig. 3). Small numbers of BrdU^+^ cells were also detected in the bronchi of both WT and Spred2^−/−^ lungs (Fig. 3).

**Figure 4:**
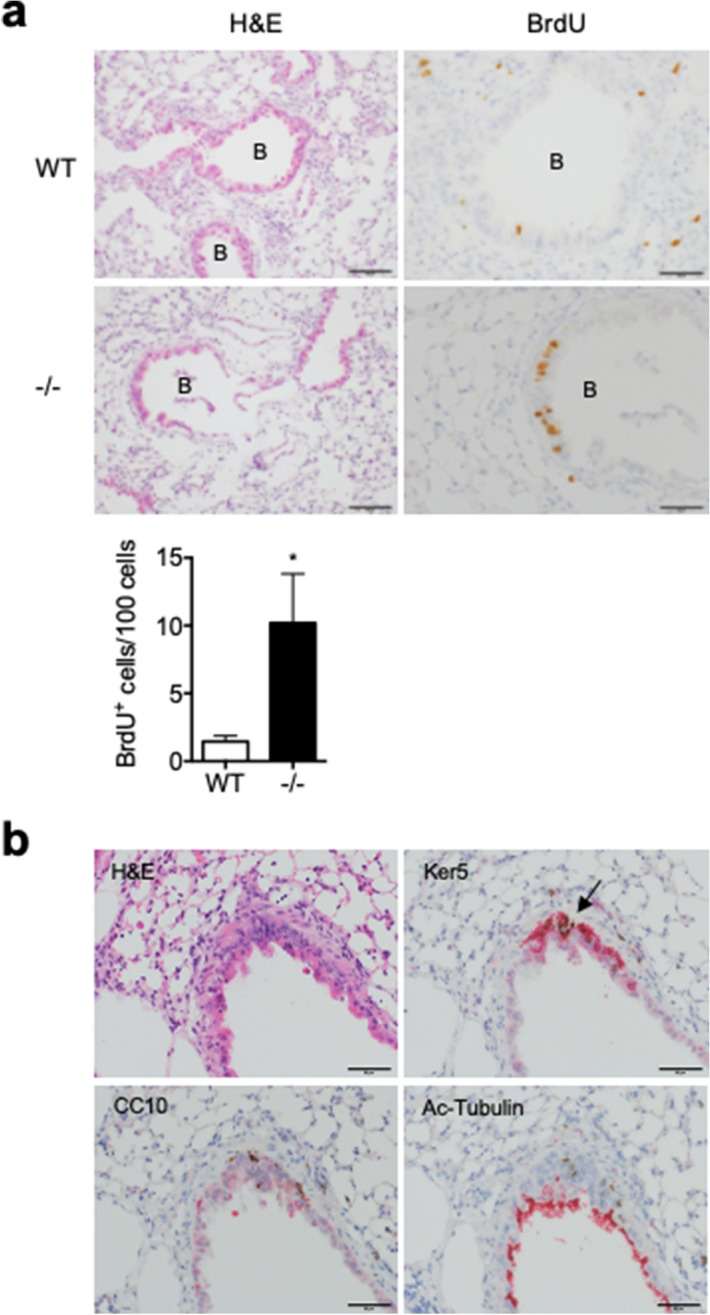
Increased proliferation of bronchial epithelial cells of Spred2^−/−^ mice 7 days after BLM administration. (**a**) Mice were injected intratracheally with 1.5 mg/kg BLM and lung tissues were harvested after 7 days. To detect the proliferation of cells, BrdU was intraperitoneally injected 2 h before the harvest. Tissue sections were subjected to H&E staining or IHC for BrdU. Representative images are shown. The original magnification was 200 × for H&E staining and 400 × for BrdU staining. The number of BrdU^+^ cells in five bronchi were counted and presented as BrdU^+^ cells/100 cells. The results are presented as mean ± SEM. n = 5. *p < 0.05. Scale bars are 100 μm for H&E staining and 50 μm for BrdU staining. (**b**) To determine cell types of BrdU^+^ cells, consecutive tissue sections were subjected to H&E staining or IHC with antibodies against keratin 5, CC10 or acetylated tubulin. The original magnification was 400×. Arrow indicates the region where several BrdU^+^ cells were found. Scale bars are 50 μm.

On day 7, a number of BrdU^+^ cells were detected in the lung of WT mice. Most of them were located in the alveolar area and only a few BrdU^+^ cells were detected among bronchial epithelial cells. By contrast, in the lung of Spred2^−/−^ mice, significantly larger numbers of BrdU^+^ cells were detected among bronchial epithelial cells mainly of large bronchi with a small number of BrdU^+^ cells of small bronchi and outside the bronchus (Fig. 4a). By double-staining, the majority of BrdU^+^ cells were positive for keratin 5 but a few of them were positive for CC10. There was no BrdU^+^ cells among acetylated tubulin-positive cells (Fig. 4b), indicating that they were basal cells and Clara cells.

**Figure 5:**
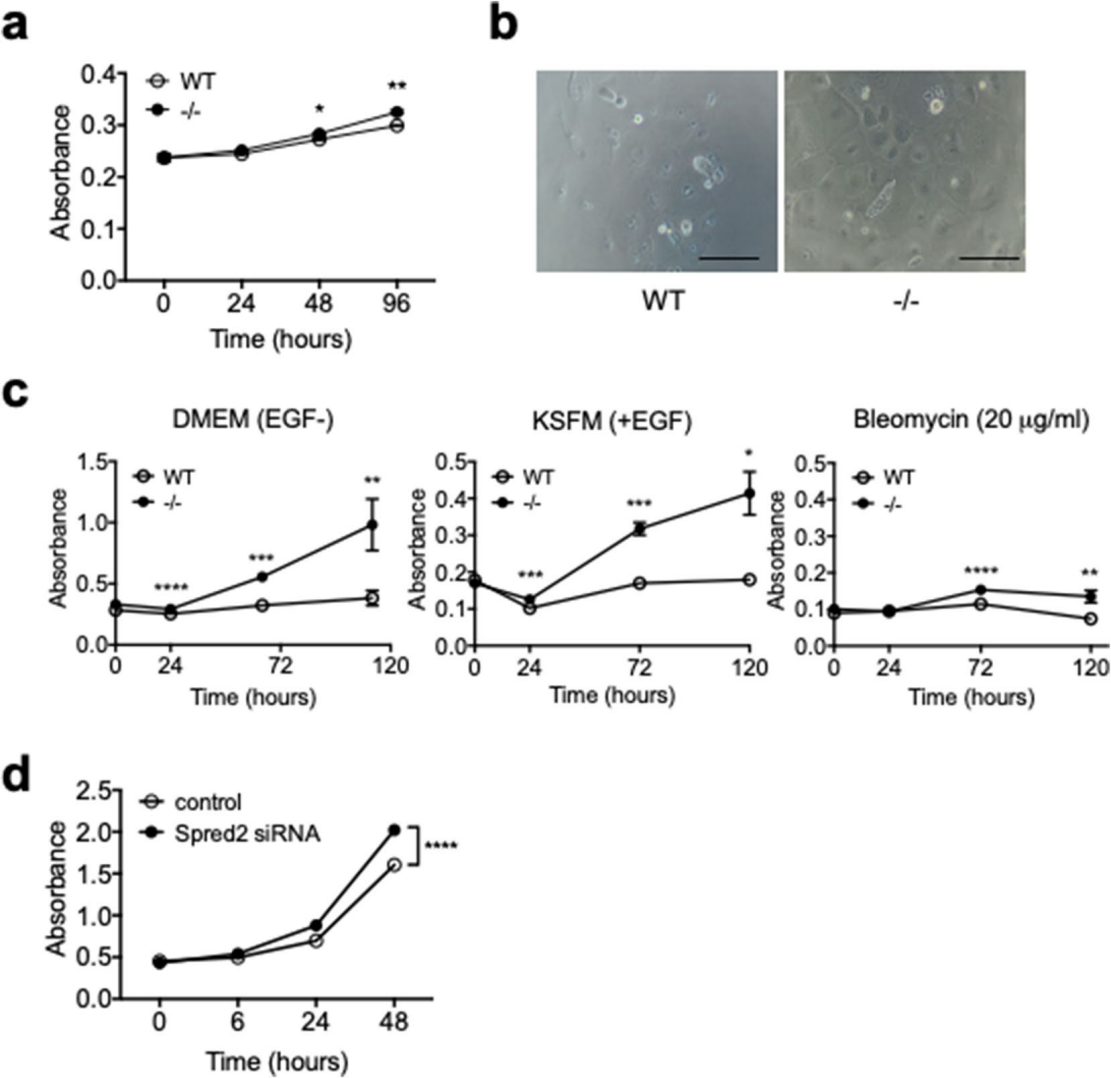
Increased proliferation of Spred2^−/−^ mouse bronchial epithelial cells in vitro. (**a**) Lung epithelial cells (LECs) were obtained from naïve WT and Spred2^−/−^ mice by negative selection with anti-CD45 Ab, followed by positive section with anti-CD326 Ab. Thirty thousand cells were seeded in 96-well plates and cultured for up to 4 days in DMEM containing 10% FBS. The growth of cells was evaluated by the CCK-8 assay. The results are presented as mean ± SEM. *p < 0.05, **p < 0.001. n = 4. (**b**) Tracheobronchial epithelial cells (TECs) were isolated from lungs of WT and Spred2^−/−^ mice as described in M&M. Five thousand cells were cultured in 96-well plastic plates in DMEM containing 10% FBS for 5 days and cells adhered to the plates were observed under inverted microscope. Representative images are presented. Scale bars are 50 μm. (**c**) Thirty thousand TECs were seeded in 96-well plates and cultured for up to 5 days in DMEM containing 10% FBS, KSFM plus EGF or KSFM plus EGF plus 20 μg/ml BLM. The growth of cells was evaluated by CCK-8 assay. The results are presented as mean ± SEM. *p < 0.05, **p < 0.001, ***p < 0.0001, ****p < 0.00001. n = 3. (**d**) MLE-12 mouse lung epithelial cell line cells were transfected with 10 pM Spred2-specific or control siRNA and the proliferation of cells was evaluated by CCK-8 assay. The results are presented as mean ± SEM. n = 12. ****p < 0.00001.

To examine the ability of lung epithelial cells to proliferate, we isolated lung epithelial cells (LECs), including both bronchial and alveolar epithelial cells, from WT and Spred2^−/−^ lungs by negative selection of CD45^−^ cells, followed by positive selection of CD326^+^ cells, and evaluated their ability to proliferate in vitro (Fig. 5a). The proliferation of Spred2^−/−^ LECs was slightly but significantly higher than that of WT LECs. We also isolated tracheal epithelial cells (TECs) and evaluated their ability to proliferate. As shown in Fig. 5b, compared with WT TECs, a larger number of Spred2^−/−^ TECs could be found on the plastic surface of 96-well plates after 5 days of culture in DMEM. By CCK-8 assay, the proliferation of Spred2^−/−^ TECs was significantly higher than that of WT TECs in both DMEM containing 10% FBS and keratinocyte serum free medium (KSFM) containing epithelial growth factor (EGF). Addition of BLM to KSFM (EGF +) impaired the proliferation of both WT and Spred2^−/−^ TECs (Fig. 5c), indicating that the direct effect of BLM on the proliferation of WT and Spred2^−/−^ TECs was similar.

Since it was difficult to culture primary epithelial cells in vitro, we analyzed the role of Spred2 in the proliferation of lung epithelial cells by knocking down the expression of Spred2 by Spred2-specific siRNA in MLE-12 cells that present the distal bronchiolar and alveolar epithelium^21^. Spred2-specific siRNA-treated cells proliferated significantly higher than cells transfected with control siRNA (Fig. 5d). These results supported the hypothesis that decreased PF detected in Spred2^−/−^ lung may be due to the increased proliferation of bronchial epithelial cells.

### Reduced BML-induced PF development in Spred2^−/−^ mice is not due to Spred2-deficiency in myeloid cells

**Figure 6:**
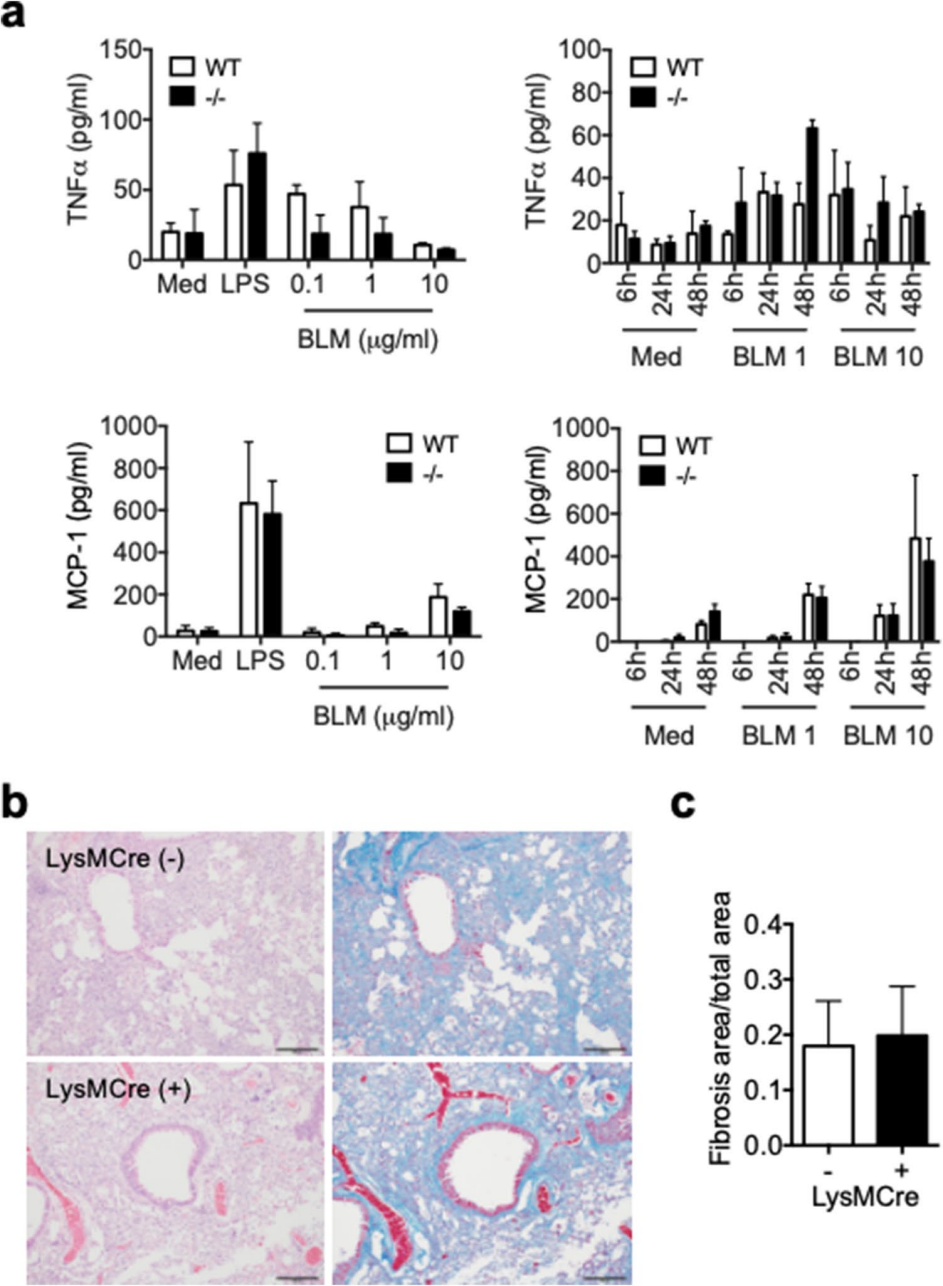
A role of macrophages in the decreased BLM-induced PF in Spred2^−/−^ mice. (**a**) One million TG-induced peritoneal macrophages were cultured in 12-well plates in the presence of different doses of BLM for different time periods or 100 ng/ml LPS for 24 h. Production of TNFα and MCP-1 was evaluated by ELISA. The results are presented as mean ± SEM. n = 3. (**b**) The development of PF was evaluated by using LysMCre^+^Spred2^F/F^ mice and LysMCre^−^Spred2^F/F^ mice. Representative images of H&E (left) and Masson’s trichrome staining (right) are shown. The original magnification was 200×. The scale bar is 100 μm. (**c**) The ratios of fibrosis area to total lung area were calculated as described in the M&M section. The results are presented as mean ± SD. n = 5 for each group.

We previously demonstrated that the production of proinflammatory mediators was up-regulated in Spred2^−/−^ resident or M-CSF-induced BM-derived macrophages^22,23^. Here, we examined the production of TNFα and MCP-1 by inflammatory macrophages obtained by intraperitoneal injection of thioglycolate (TG) in response to BLM or LPS. BLM was previously reported to induce cytokine production in macrophages^24^. Upon injury to lung epithelial cells, translocated bacteria and injured host cells could release ligands for toll-like receptor 4 (TLR4), such as LPS and high mobility group box 1, and these TLR4 ligands could activate macrophages and induce the production of proinflammatory cytokines. As shown in Fig. 6a, WT and Spred2^−/−^ inflammatory macrophages produced similar levels of TNFα or MCP-1 in response to LPS after 24-h incubation. They also produced similar levels of TNFα or MCP-1 in response to BLM with similar kinetics.

To obtain additional evidence that Spred2-deficiency in myeloid cells, such as macrophages, is not responsible for the reduced PF development in response to BLM, we examined the development of PF in myeloid cell-specific Spred2-deficient mice (LysMCre^+^Spred2^F/F^). We first generated Spred2-floxed mice (Spred2^F/F^) by inserting two *loxP* sites flanking exon 6 of the *Spred2* gene and then crossed them to LysMCre mice (Supplementary Fig. S2a). Deletion of exon 6 in the bone marrow cells of the conditional Spred2-deficient mice was confirmed by PCR (Supplementary Fig. S2b) As shown in Fig. 6b,c, similar levels of collagen deposition were present in the lung of both LysMCre^+^Spred2^F/F^ and control LysMCre^−^Spred2^F/F^ mice as indicated by Masson’s trichrome staining. These results indicate that the Spred2-deficiency in myeloid cells, such as macrophages, was not responsible for the reduced BLM-induced PF development in Spred2^−/−^ mice.

### Increased proliferation of fibroblasts in the absence of Spred2 in vitro

**Figure 7:**
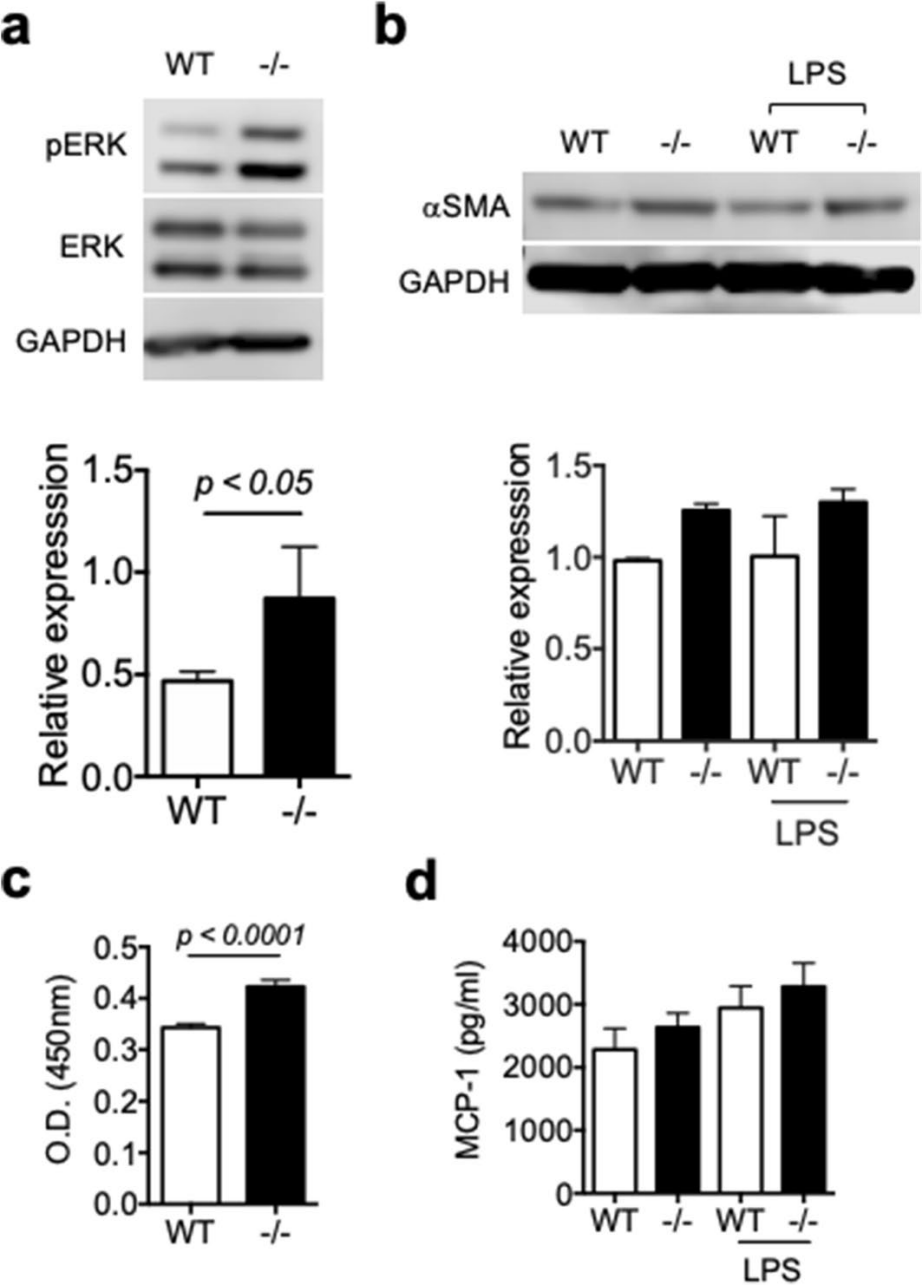
Increased proliferation of Spred2^−/−^ fibroblasts in vitro. Lung fibroblasts were obtained from WT and Spred2^−/−^ mice and cultured in vitro. (**a**) The phosphorylation of ERK was examined by western blotting. Representative photos of the blots are presented. The results of estern blotting were also quantitated by using the ImageJ software. The results are presented as mean ± SD. n = 3. (**b**) The expression of αSMA was examined by western blotting with or without LPS-stimulation. The results of western blotting were also quantitated by using the ImageJ software. The results are presented as mean ± SD. n = 2. (**c**) The proliferation of lung fibroblasts was examined by MTT assay. The results are presented as mean ± SEM. n = 12. (**d**) The production of MCP-1 by lung fibroblasts was examined by ELISA. The results are presented as mean ± SD. n = 3.

We next examined fibroblasts. Lung fibroblasts were obtained from the lung of WT and Spred2^−/−^ mice and propagated in vitro. As shown in Fig. 7a, the phosphorylation of ERK was constitutively increased in Spred2^−/−^ fibroblasts. The level of αSMA tended also higher in Spred2^−/−^ fibroblasts but the difference was not statistically significant. The stimulation with the TLR4 ligand LPS did not affect its level (Fig. 7b). Spred2^−/−^ fibroblasts proliferated significantly faster than WT fibroblasts without any additional stimuli by MTT assay (Fig. 7c). The production of MCP-1 in the absence or the presence of LPS was similar between WT and Spred2^−/−^ fibroblasts (Fig. 7d). Thus, Spred2-deficiency in fibroblasts did not appear to account for the reduced BLM-induced PF development in Spred2^−/−^ mice.

### Accumulation of Spred2 mRNA in bronchial epithelial cells of BLM-treated WT mice

**Figure 8:**
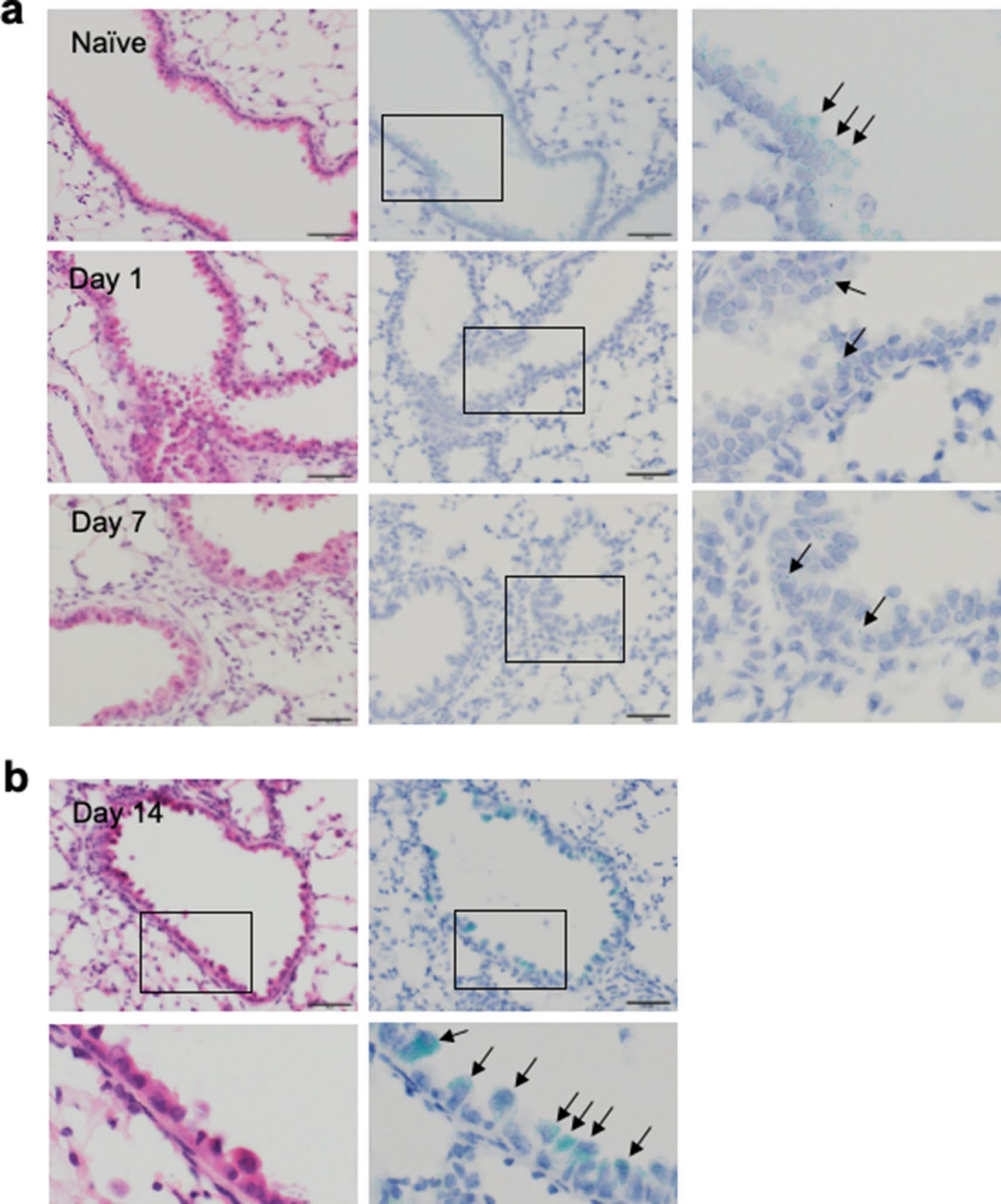
Expression of Spred2 mRNA in the lung of WT mice before and after BLM treatment. Lungs were harvested from untreated WT mice (naïve) or 1, 7 or 14 days after the intratracheal instillation of BLM. (**a**) The expression of Spred2 mRNA was examined on day 0 (naïve), 1 and 7 after BLM treatment by ISH. Left panels: H&E staining. Middle panels: ISH. Right panels: The area indicated by a rectangle in the middle panel is further magnified. Almost all cells expressed a significant level of Spred2 mRNA (blue-green dots) in naive mice as indicated by arrows. By contrast, Spred2 mRNA was expressed at a low level on day 1 and 7 as indicated by an arrow. The magnification of the original photos was 400×. The scale bars are 50 μm. (**b**) The expression of Spred2 mRNA on day 14 was examined by ISH. Top left panel: H&E staining. Bottom left panel: The area indicated by a rectangle in the top left panel is further magnified. Top right panel: ISH. Bottom right panel: The area indicated by a rectangle in the top right panel is further magnified. Spred2 mRNA accumulated in approximately 50% of bronchial epithelial cells, likely Clara cells by morphology, as indicated by arrows. The magnification of the original photos was 400×. The scale bars are 50 μm.

To obtain additional evidence supporting the hypothesis that Spred2-deficiency in bronchial epithelial cells was responsible for the alleviated PF in Spred2^−/−^ mice, we examined the expression of Spred2 in the mouse lung. We attempted to detect Spred2 protein by IHC using several antibodies, but our attempts were not successful. Therefore, we used in situ hybridization (ISH) to detect Spred2 mRNA. As shown in Fig. 8a,b, significant levels of Spred2 mRNA (blue-green dots) could be detected in almost all bronchial epithelial cells, but not in alveolar epithelial cells, of untreated WT mice. The expression levels appeared to decrease on day 1 and day 7 after BLM administration. Interestingly, however, strong accumulation of Spred2 mRNA was observed in approximately 50% of bronchial epithelial cells with a distinctive dome-shaped luminal surface, characteristic of Clara cells^25^. These findings strongly support the role of Spred2 in the proliferation of bronchial epithelial cells and tissue repair in this model.

## Discussion

BLM-induced cell toxicity occurs in various organs, such as the lung and skin, and mucous membranes, due to the lack of BLM hydrolase, the enzyme that inactivates BLM. Delivery of BLM to the lung causes pulmonary injury, inflammation, and subsequent fibrosis^8^. Spred2 is an endogenous inhibitor of the Ras/Raf/MEK/ERK pathway involved in a variety of cellular processes, including cell proliferation and inflammation^26^. We previously examined the role of Spred2 in lung injuries by using two acute injury models; LPS-induced ALI and ischemia–reperfusion injury models^19,20^. In both studies, lung injury was exacerbated in the absence of Spred2 with increased inflammatory responses. These results led us to the hypothesis that Spred2-deficiency may enhance PF development induced by the administration of BLM. Contrary to our hypothesis, however, the development of PF was alleviated in Spred2^−/−^ mice compared to WT mice. This was associated with an increase in bronchial epithelial cell proliferation. Furthermore, the proliferation of Spred2^−/−^ LECs and TECs or MLE-12 cells transfected with Spred2-specific siRNA was higher than that of control cells in vitro. By ISH, Spred2 mRNA was detected almost exclusively in bronchial epithelial cells. These results strongly suggested that Spred2-deficiency alleviates BLM-induced PF via increased proliferation of bronchial epithelial cells that reduces subsequent development of PF by promoting the healing of injured lung tissues. To our knowledge, this is the first study to demonstrate a role of Spred2 in PF.

The bronchial epithelium protects the internal milieu of the lung by forming a physical barrier involving adhesive complexes and a chemical barrier involving the secretion of mucus, and most environmental challenges are largely overcome without the need to develop inflammatory responses^27^. The injury to bronchial epithelial cells is followed by an inflammatory phase characterized by the infiltration of leukocytes, increased vascular leak and upregulation of pro-inflammatory cytokines and chemokines. In LPS-induced ALI model, lung injury was exacerbated with increased inflammatory responses in Spred2^−/−^ mice^19^. LPS injected into the trachea can directly activate TLR4 on bronchial epithelial cells and alveolar macrophages to produce proinflammatory cytokines and chemokines, whereas BLM damages epithelial cells and the induction of the inflammatory response is secondary to the damage to epithelial injury. Thus, the mechanisms of lung injuries caused by intratracheal injection of LPS or BLM are different, and this may explain the reason why the two models provided quite opposite outcomes.

During the development of PF, lung macrophages produce a variety of cytokines critical for the process^28^. We found that the production of the proinflammatory cytokine TNFα and the chemokine MCP-1 in response to BLM or LPS by Spred2^−/−^ inflammatory macrophages was similar to that by WT macrophages, suggesting that Spred2-deficiency in macrophages is not responsible for the decreased level of PF in Spred2^−/−^ mice. When myeloid cell-specific Spred2-deficient mice were subjected to BLM, they developed similar levels of PF to that in control mice. Thus, Spred2-deficiency in myeloid cells does not account for the decreased PF detected in systemic Spred2^−/−^ mice.

Fibroblasts are another candidate cell population that contributes to the development of PF. Epithelial cell dysfunction or damages can lead to the activation of fibroblasts and the deposition and remodeling of matrix. The myofibroblast is the classic pathologic fibroblast phenotype described in IPF lungs^2,29^. In the present study, lung fibroblasts isolated from Spred2^−/−^ mice showed an increased level of constitutive ERK phosphorylation and proliferation compared to those from WT mice. They also produced a high level of MCP-1 with or without stimulation. Thus, Spred2^−/−^ fibroblasts appear to be more myofibroblast-like in the in vitro culture. On the other hand, the degree of fibrosis was lower and the number of BrdU^+^ fibroblasts were not increased in the lung of BLM-treated Spred2^−/−^ mice in vivo, showing a reverse behavior of Spred2^−/−^ fibroblasts in the in vitro culture and in the tissue. These observations strongly suggest that Spred2-deficiency in fibroblasts did not contribute to the alleviated PF in Spred2^−/−^ mice.

The phenotype of Spred2^−/−^ mice in BLM-induced PF model is similar to that detected in an acute colitis model induced by dextran sulfate sodium (DSS)^30^. In the colitis model, DSS damages colonic epithelial cells, resulting in the translocation of bacteria followed by activation of cells in mucosa and induction of inflammatory responses. Colonic epithelial cells of DSS-treated Spred2^−/−^ mice exhibited increased proliferation and healing compared to those of WT mice, likely resulting in decreased levels of acute colitis. These findings suggest interesting dual roles of Spred2; the loss of Spred2 in epithelial cells is anti-inflammatory whereas the loss of Spred2 in leukocytes, such as macrophages, is proinflammatory. The role of Spred2 in neuro-regeneration was studied in the dorsal telencephalon followed by mechanical-lesion using zebrafish. Interestingly, the expression of Spred2 was significantly reduced in the telencephalon post-lesion and gradually increased to normal levels, suggesting that a decrease in spred2 level after injury might be associated with activation of the ERK pathway to stimulate cell proliferation in the adult zebrafish brain^31^. The expression of Spred2 mRNA also decreased in whole lungs of BLM-treated WT mice on day 7 and 14 by qRT-PCR (Supplementary Fig. S3). A similar decrease was observed in bronchial epithelial cells on day 1 and 7 by ISH, but strong accumulation of Spred2 mRNA was detected in a portion of epithelial cells, likely Clara cells. These findings suggest that the expression of Spred2 may be altered during the host response to a damage to bronchial epithelial cells. The role of Spred2 in cell proliferation was also found in other mouse disease models^32,33^. In the meantime, it remains unclear why increased BrdU^+^ cell population in BLM-treated Spred2^−/−^ mice was basal cells of large bronchi, but not of Clara cells. Additional studies using conditional Spred2^−/^ mice in which Spred2 is deficient in basal cells or Clara cells will be important to better understand the role of Spred2 in the proliferation of these cell types. It is also important to validate the data obtained by ISH at a protein level when a reliable antibody against mouse Spred2 becomes available.

We recently obtained human lung tissues from lung cancer patients with significant PF in the background, and isolated fibroblasts from non-fibrotic and fibrotic lesions of each case. Our preliminary results obtained by qRT-PCR suggest that the expression of Spred2 mRNA by fibroblasts from the fibrotic lesions of UIP cases was significantly increased (data not shown). It is unclear what this means at present, but it is possible that the expression of Spred2 mRNA is dysregulated in those cells. Unlike in the mouse model, PF in the patients was more advanced and we cannot compare the results obtained from the mouse model and human patients. More detailed studies, including the expression of Spred2 in epithelial cells, are necessary to understand the role of Spred2 in the development of human PF.

In conclusion, we have identified a novel role of Spred2 in the development of PF after injury by BLM in mice. Unlike previous reports demonstrating that endogenously expressed Spred2 negatively regulates acute inflammatory responses by downregulating the production of proinflammatory cytokines, downregulation of Spred2 may be beneficial for the recovery of the epithelium. Thus, this possibility should be considered when Spred2-mediated therapy is proposed to treat patients with PF and other inflammatory diseases.

## Materials and methods

### Reagents

Fetal bovine serum (FBS) was from HyClone (Logan, UT). KSFM was from Gibco (Grand Island, NY). Antibodies against p44/42 MAPK (ERK1/2), phospho-p44/42 MAPK (ERK1/2) and GAPDH were from Cell Signaling Technology (Danvers, MA). DMEM, LPS (011B4) and BrdU were from Sigma-Aldrich, St. Louis, MO. Rat monoclonal Ab against Ly6G (clone 1A8) and rabbit polyclonal Ab against keratin 5 were from BioLegend (San Diego, CA). Rabbit polyclonal antibodies against αSMA or CC10 were from Proteintech (Rosemont, IL). Mouse monoclonal Ab against acetylated tubulin (clone 6-11B-1) was from Sigma Aldrich. Rat monoclonal Ab against BrdU (clone ICR1) was from GeneTex (Irvine, CA). BLM (BLM-AP302) was from Enzo Life science (Farmingdale, NY). PCR primers for *Spred2* (Mm 01223872_g1) (Hs 00986220_m1), *Fn1* (Mm 01256744_m1) (Hs 00365052_m1), *Vim* (Mm 01333430_m1) (Hs 00185584_m1), *Cdh1* (Mm01247357_m1), *Col1a1* (Mm00801666_g1), *TNFα* (Mm00443258_m1), *TGF-β1* (Mm 01178820_m1), *Mcp-1/Ccl2* (Mm 00441242_m1) were from Applied Biosystems (Foster City, CA).

### Mice

The generation of Spred-2^−/−^ mice on the C57BL/6J background was previously reported^15^. C57BL/6J mice were used as WT mice. The absence of *Spred2* mRNA expression in the lung of Spred2^−/−^ mice was confirmed by RT-qPCR.

To generate Spred2^F/F^ mice, an approximately 11-kb fragment of mouse genomic DNA spanning the exon 5 and 6 of the Spred2 gene was retrieved from a mouse BAC clone into pLMJ235 vector containing the thymidine kinase gene. A loxP site and a neo cassette flanked by two loxP sites were inserted downstream and upstream of exon 6 by using a recombinogenic cloning method. The targeting vector was then electroporated into C57BL/6 mouse ES cells and Spred2 flox-neo mice were generated. To generate Spred2 floxed mice (Spred2^F/F^), Spred2 flox-neo mice were crossed to CAG-Flpe mice on a C57BL/6 background^34^ (RIKEN Bioresource Center, Tsukuba, Japan). Heterozygous mice (Spred2^F/+^) were mated to generate homozygous Spred2 floxed mice (Spred2^F/F^) (Supplementary Fig. S2a). Spred2^F/F^ mice were then crossed to LysMCre on a C57BL/6 genetic background^35^ to generate myeloid cell- specific Spred2^−/−^ mice (Supplementary Fig. S2b).

All mice were bred and maintained under a continuous 12 h light: 12 h dark cycle in a specific pathogen-free condition at the Department of Animal Resources, Okayama University (Okayama, Japan). Male mice (8-week-old) were used in this study. Mice were fed a standard laboratory diet and water ad libitum. The Animal Care and Use Committee at Okayama University approved all animal experiments conducted in this study (OKU-2013348, OKU-2018064), and all experiments were performed in accordance with relevant guidelines and regulations.

### Bleomycin-induced pulmonary fibrosis model

Mice were anesthetized with intraperitoneal injection of sodium pentobarbital, followed by ketamine HCl, and intratracheally injected with BLM dissolved in PBS (1.5 mg/kg). At 1, 7 and 14 days after BLM administration, mice were euthanized. The left lobe of the lung was fixed with neutral buffered formalin and embedded in paraffin; 4-µm paraffin sections were made and stained with H&E or Masson’s trichrome staining. The tissue was outlined by the freehand selection tool and fibrotic area was measured by using the ImageJ 1.45. according to the method by A. Onoda (https://www.slideshare.net/mobile/atsutoonoda/image-j-52335489). The right lobe was immediately frozen in liquid nitrogen and used for RT-qPCR and ELISA. To examine cell proliferation in vivo, mice were intraperitoneally injected with BrdU. Two hours later, mice were euthanized and the incorporation of BrdU was examined by IHC.

### Isolation of lung epithelial cells (LECs)

Mouse LECs were isolated from non-treated mice using a previously described method with modifications^36^. In brief, lungs were removed from untreated WT or Spred2^−/−^ mice. They were minced in a gentleMACSTM Dissociator (Miltenyi Biotec, Gladbach, Germany) and digested in 5 ml Hank’s balanced salt solution (HBSS) containing 20 μg/ml collagenase (Wako, Japan), 150 U/ml DNase I (Roche, Basel, Switzerland) and 20 mM HEPES (Gibco), for 30 min at 37 °C with occasional shaking. The cell suspensions were filtered through 100-μm nylon mesh, and centrifuged at 400 × g for 6 min. The cells were washed once with PBS and then incubated with anti-CD45 microbeads. CD45^+^ cells were removed over MS MiniMACS separation columns (Miltenyi Biotec), and CD45^−^ cells were collected and incubated with biotin-labeled anti-CD326 Ab (Biolegend, USA), followed by addition of streptavidin-coated microbeads. Magnetic beads-labeled CD326^+^ cells were collected on a separation column, suspended in DMEM high glucose medium containing 10% FBS. CD326^+^ cells included bronchial and alveolar epithelial cells.

### Isolation of tracheobronchial epithelial cells (TECs)

Tracheobronchial epithelial cells (TECs) were isolated using a previously described method with modifications^37,38^. Briefly, tracheal tissues from 6 WT or Spred2 KO mice were placed into a 50 ml conical tube containing 30 ml DMEM containing penicillin and streptomycin on ice. In a sterile lamellar flow hood, tracheal tissues were transferred to a sterile 100 mm petri dish containing 10 ml DMEM with antibiotics. Connective tissues were gently dissected with sterile forceps and surgical scissors. Tracheal tissues were transferred to a new 100 mm petri dish containing 10 ml DMEM containing antibiotics to rinse. Tracheas were cut along vertical axis to expose lumen, and then transferred to a 50 ml tube containing 10 ml DMEM with 0.15% pronase and antibiotics (pronase solution). After overnight incubation at 4 °C, the tubes were gently rocked 10–12 times, and then let stand for 30–60 min at 4 °C. Ten ml DMEM containing 20% FBS and antibiotics was added to each tube and the tubes were rocked 12 times. Tracheas were removed from pronase solution, and this solution was set aside on ice. Tracheas were transferred to a conical tube containing DMEM and the tubes were inverted 12 times. This process was repeated two more times. Pronase solution was combined with the three supernatants from the previous step into one 50 ml tube, and the remaining tissues were discarded. The 50 ml tubes were centrifuge at 1400 rpm (390 × g) for 10 min at 4 °C, and the supernatants were discarded. The cell pellets were gently resuspended in 1 ml DNase I solution (100–200 μl/trachea), incubated for 5 min on ice and centrifuged at 1400 rpm (390 × g) for 5 min at 4 °C. The cell pellet was resuspended in 8 ml DMEM containing 10% FBS, plated on petri dish, incubated at 37 °C in a CO_2_ incubator for 5 h (Note: this is a negative selection step for fibroblasts), and then nonadherent cells (cell suspension) were collected from the dishes. The dishes were rinse twice with 4 ml DMEM containing 10% FBS. Cell suspension and washes were pooled together in a 50 ml conical tube, centrifuged and resuspended in DMEM containing 10% FBS. The concentration of cells was examined by trypan blue staining.

### Isolation of lung fibroblasts

Lung fibroblasts were isolated from non-treated mice, as previous described^39^. In brief, lung tissues were isolated under sterile condition, and 1 mm^3^ fragments were placed on tissue culture plates containing high glucose DMEM with 15% FBS and then incubated at 37 °C in a 15% CO_2_ atmosphere. After 5-day culture, lung tissues were removed from the plates and fibroblasts were grown to 80% confluence, and then transferred to 175 mm^2^ flasks. Fibroblasts were then harvested and used for RT-qPCR, MTT assay and western blotting.

### Short-interfering (si) RNA

One million MLE-12 cells (ATCC, Manassas, VA) were transfected with 2 μg Spred2-specific or control siRNA (Thermo Scientific, Yokohama, Japan) using the Amaxa nucleofector kit V (Lonza Cologne AG, Cologne, Germany) according to the manufacturer’s instructions, and plated in a 24-well plate. The siRNA efficacy was validated by real-time qRT-qPCR. The expression of Spred2 was routinely 30% or less of the levels detected in control cells.

### Real-time quantitative PCR (RT-qPCR)

Total RNA was isolated from cultured cells or whole lungs using the High Pure RNA Isolation Kit or High Pure RNA Tissue Kit (Roche Applied Science, Mannheim, Germany), respectively. First-strand cDNAs were synthesized from total RNA using the High capacity cDNA reverse transcription kit (Applied Biosystems, Foster City, CA, USA), and used as templates for PCR. RT-qPCR analysis was performed using the StepOnePLus Real-Time PCR system with Taqman PCR master mix (Applied Biosystems). The level of gene expression was normalized using the level of GAPDH expression as an internal control, and relative fold change values were calculated based on unstimulated or WT control group that were assigned an arbitrary value of 1.

### ELISA

The concentrations of murine cytokines were measured using a standard sandwich ELISA method, as previously described^40^. ELISAs used in this study did not cross-react with other known murine cytokines. For lung cytokine measurements, lungs were homogenized in PBS containing 0.1% TritonX-100 and complete protease inhibitor (Roche) and centrifuged; the cleared supernatants were harvested and used for ELISAs.

### Western blotting

Cells or lung tissues were lysed in a lysis buffer (Cell Signaling), briefly sonicated, incubated on ice for 30 min, and then centrifuged at 12,000×*g* for 10 min. Supernatants were collected and stored at – 30 °C until use. For protein fractionation, 10 µg of each cell lysate was loaded on gels and proteins were fractionated by sodium dodecyl sulfate (SDS)-polyacrylamide gel electrophoresis (Life Technologies) and transferred onto nitrocellulose membranes. After overnight incubation with a primary antibody, the membranes were washed and incubated with horseradish peroxidase-conjugated anti-rabbit or -mouse IgG secondary antibody (Santa Cruz Biotechnology, Santa Cruz, CA, USA). The presence of proteins of interest was visualized and quantitated with C-DiGit Blot scanner (Scrum, Tokyo, Japan). Blots were photographed, digitized and the density of each band was analyzed using Image J, a public domain software developed by the NIH.

### Detection of apoptotic cells

Apoptotic cells in the lung were detected by TUNEL assay using the in situ apoptosis detection kit (TACS2 TdT-DAB; Trevigen, Gaithersburg, MD) according to the manufacturer’s instructions except that hematoxylin was used for counterstaining. The number of positive cells was counted under microscope.

### Immunohistochemistry (IHC)

Serial sections of 4-μm thickness were prepared from formalin-fixed and paraffin-embedded blocks. One section was stained with H&E and others were used for immunohistochemistry for each block. Immunostaining was performed manually by a conventional method as previously reported^41^. Briefly, sections were deparaffinized in xylene and rehydrated in a sequence of descending concentrations of ethanol. Endogenous peroxidase reactivity was blocked with 3% H_2_O_2_ for 10 min. Sections were enclosed in a pressure cooker with citrate buffer (pH6.0) and microwaved (500 W) continuously for 20 min for antigen retrieval, and then incubated with anti-BrdU (1:50) or anti-Ly6G (1:100) antibody for 1.5 h at room temperature. After washing, they were treated with peroxidase-labeled anti-rat IgG according to the manufacturer’s instructions. Histochemical reactions were developed using 3, 3-diaminobenzidine (DAB) as the chromogenic substrate for peroxidase. Finally, sections were counterstained with hematoxylin, dehydrated, and mounted. For double immunostaining, after antigen retrieval, sections were first treated with antibodies against keratin 5 (1:500), CC10 (1:500) or acetylated tubulin (1:200), followed by alkaline phosphatase-labeled secondary antibodies and histochemical reactions were developed using Vector Red. The additional immunohistochemistry for BrdU was started by antigen retrieval, followed by primary and secondary antibody incubation, and completed with histochemistry using DAB as a chromogen.

### In situ hybridization (ISH)

Expression of mouse Spred2 mRNA in lung tissues was detected by RNAScope 2.5 Duplex Detection Kit (ACD, Inc., Hayward, CA) according to the direction provided by the supplier. Briefly, tissue sections in 5-μm thickness were deparaffinized in xylene, followed by rehydration in a sequence of descending concentrations of ethanol. Tissue sections were then incubated in Target Retrieval Reagent maintained at a boiling temperature (98 °C) using a hot plate for 20 min, rinsed in deionized water, dried and then treated with protease at 40 °C for 40 min in a Dako StatSpin Hybridizer (Agilent, Santa Clara, CA). Tissue sections were then incubated at 40 °C in a hybridization buffer containing target probes (Mm-Spred2-C1, # 527581-C1) for 3 h. After the hybridization step, slides were washed with wash buffer three times at room temperature, and then multiple signal amplification molecules were hybridized. Chromogenic detection was performed using FastGreen, followed by counterstaining with hematoxylin^42^. Images were acquired using an Olympus BX43 light microscope connected to a DP73 digital camera (Olympus, Tokyo, Japan).

### Cell proliferation assay

Thirty thousand LECs in DMEM containing 10% FBS or TECs in DMEM containing 10% FBS, KSFM containing EGF (5 ng/ml) or KSFM containing EGF plus BLM or MLE-12 cells in DMEM containing 10% FBS were seeded in 96-well plate and cultured for up to 5 days. The growth of cells was evaluated by the CCK-8 assay (Dojindo, Kumamoto, Japan). For fibroblasts, 5 × 10^3^ cells in DMEM containing 10% FBS were cultured in 96 well plates for 4 days and the proliferation of cells was evaluated by MTT assay (Sigma-Aldrich).

### Statistics

Data were analyzed and plotted by using the GraphPad Prism 6.0e (GraphPad Software, San Diego, CA). The statistical significance of differences between data sets was evaluated using the Student’s *t*-test. *p* < 0.05 was considered to represent a statistically significant difference.

## Supplementary information

Supplementary Figures.

Supplementary Figure.
