# Supplementary material for: Spred2-deficiency enhances the proliferation of lung epithelial cells and alleviates pulmonary fibrosis induced by bleomycin
Source: Sci Rep. 2020 Oct 5;10:16490. doi: 10.1038/s41598-020-73752-3 (PMC7536438; doi:10.1038/s41598-020-73752-3)
Supplement: Supplementary file 1 — Supplementary Figures. [file 41598_2020_73752_MOESM1_ESM.pdf]

Supplementary Figures

Spred2-Deficiency Enhances the Proliferation of Lung Epithelial Cells and Alleviates Pulmonary Fibrosis Induced by Bleomycin

Akina Kawara[1], Ryo Mizuta[1], Masayoshi Fujisawa[1], Toshihiro Ito[2], Chunning Li[1], Kaoru Nakamura[1], Cuiming Sun[1], Masaki Kuwabara[1], Masahiro Kitabatake[2], Teizo Yoshimura[1] & Akihiro Matsukawa[1]

[1] Department of Pathology and Experimental Medicine, Graduated School of Medicine, Dentistry and Pharmaceutical Sciences, Okayama University, 2-5-1 Shikata, Kita-ku, Okayama, 700-8558, Japan.

[2] Department of Immunology, Nara Medical University, Kashihara, 634-8521, Japan.

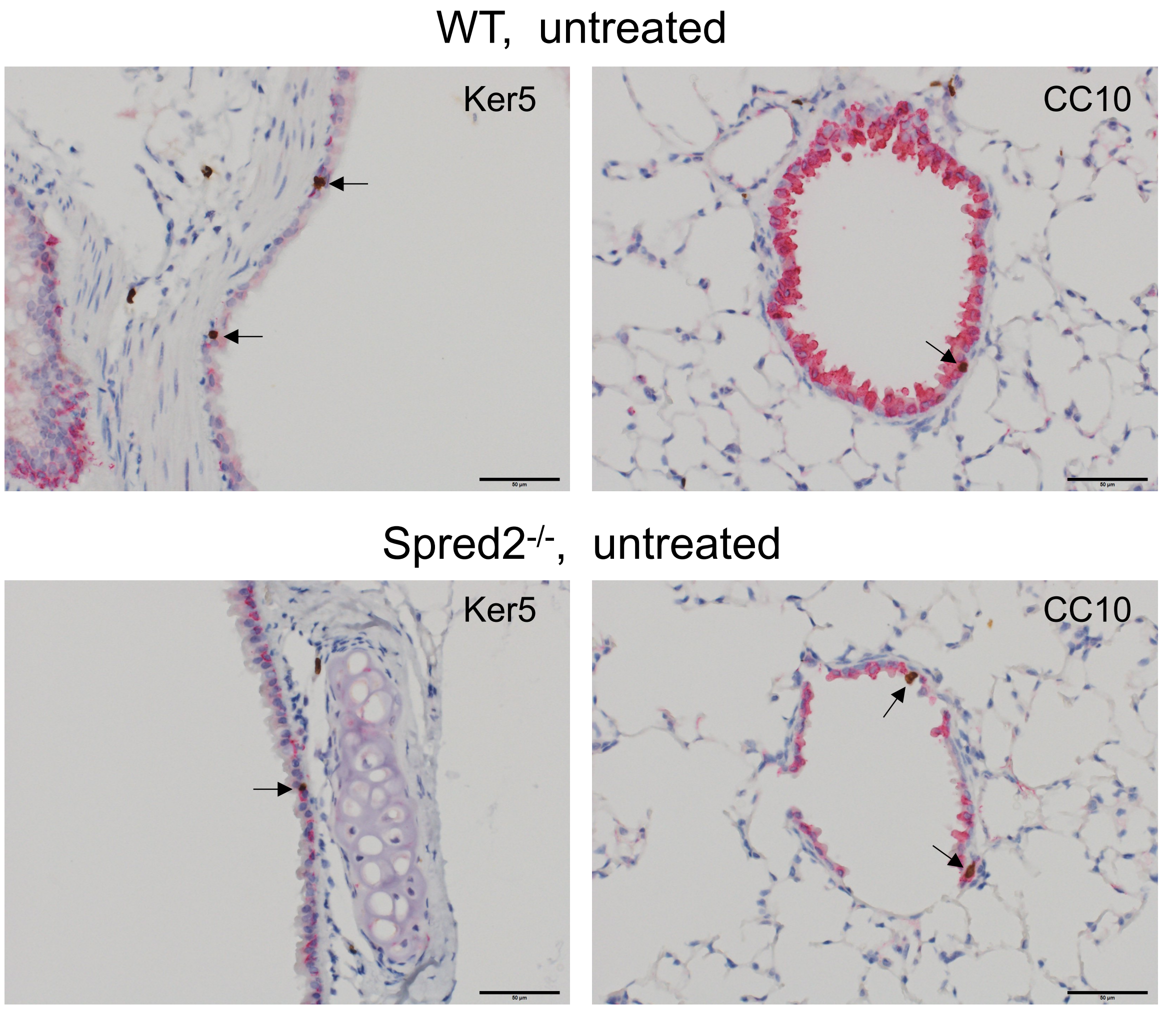

Supplementary Figure S1. Incorporation of BrdU by Keratin 5- and CC10-positive bronchial epithelial cells of untreated WT and spred2$^{-/-}$ mice. Paraffin sections of lungs from WT or Spred2$^{-/-}$ mice were double stained for BrdU and Keratin 5 or CC10 as described in the M&M section. Arrows indicate double positive cells. Scale bars are 50 µm.

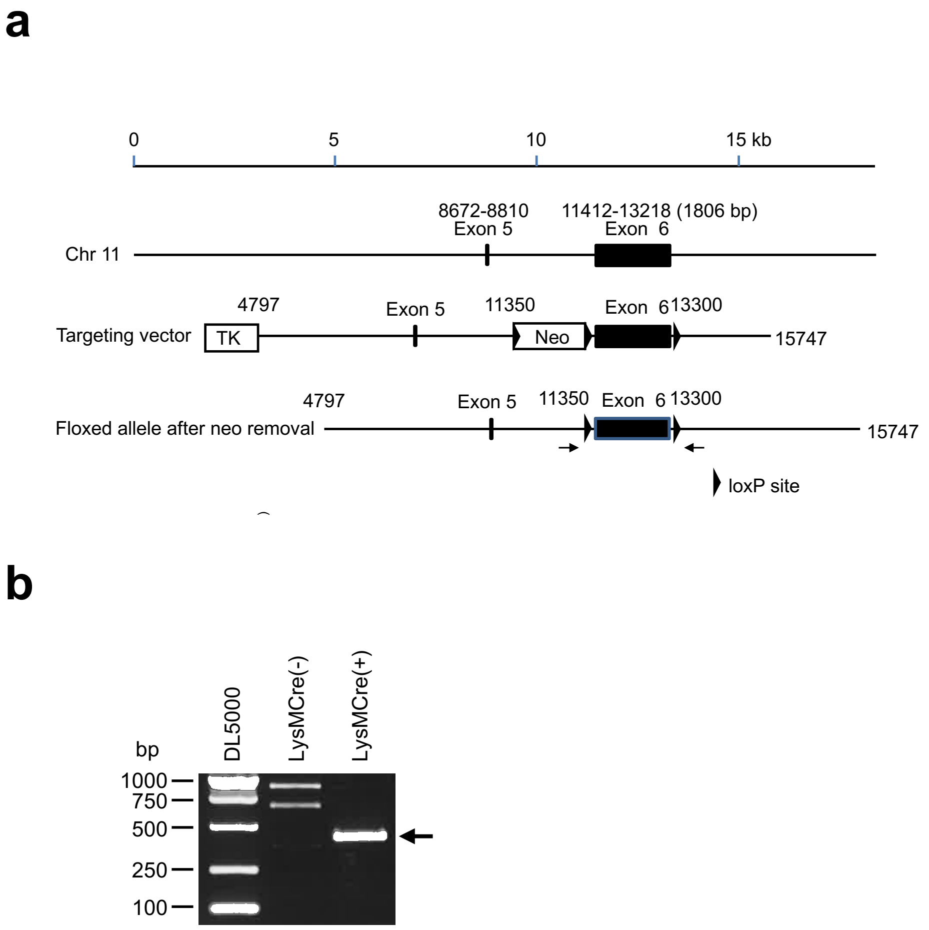

Supplementary Figure S2. Generation of myeloid cell-specific Spred2-deficient mice. **a.** To generate Spred2$^{flox/flox}$ mice, an approximately 11-kb fragment of mouse genomic DNA spanning the exon 5 and 6 of the Spred2 gene was retrieved from a mouse BAC clone into pLMJ235 vector containing the thymidine kinase gene. A loxP site was inserted upstream of exon 6 and a neo gene cassette flanked by loxP-FRT was inserted downstream of exon 6 by using a recombinogenic cloning method. The targeting vector was then electroporated into C57BL/6 mouse ES cells and Spred2 flox-neo mice were generated. To generate Spred2 floxed mice (Spred2$^{F/F}$), Spred2 flox-neo mice were crossed to CAG-Flpe mice on a C57BL/6 background (RIKEN Bioresource Center, Tsukuba, Japan). Heterozygous mice (Spred2$^{F/+}$) were mated to generate homozygous Spred2 floxed mice (Spred2$^{F/F}$). Two arrows indicate the locations of 2 primers used to detect the knockout allele. **b.** Spred2$^{F/F}$ mice were crossed to LysMCre on a C57BL/6 genetic background to generate myeloid cell- specific Spred2$^{-/-}$ mice. The presence of the knockout allele in the bone marrow cells of LysMCre^{+}Spred2$^{F/F}$ mice was confirmed by PCR. The 400-bp band (arrow) indicates the knockout allele.

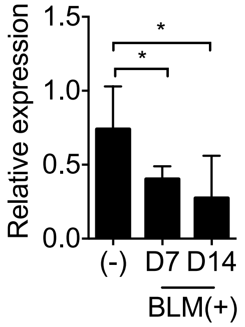

Supplementary Figure S3. Downregulation of Spred2 mRNA expression in the lung of WT mice after administration of BLM. Lungs were obtained from untreated and BLM (1.5 mg/kg)-treated WT mice on Day 7 and 14, and the levels of Spred2 mRNA were examined by RT-qPCR. The results are presented as mean ± SD. n = 3 (for untreated), 8 (for Day 7) and 7 (for Day 14). *$p < 0.05$.
