# Supplementary material for: Spred2-deficiency enhances the proliferation of lung epithelial cells and alleviates pulmonary fibrosis induced by bleomycin
Source: Sci Rep. 2020 Oct 5;10:16490. doi: 10.1038/s41598-020-73752-3 (PMC7536438; doi:10.1038/s41598-020-73752-3)
Supplement: Supplementary file 2 — Supplementary Figure. [file 41598_2020_73752_MOESM2_ESM.pdf]

Supplementary Information (Original photos used for Figure 6)

Spred2-Deficiency Enhances the Proliferation of Lung Epithelial Cells and Alleviates Pulmonary Fibrosis Induced by Bleomycin

Akina Kawara[1], Ryo Mizuta[1], Masayoshi Fujisawa[1], Toshihiro Ito[2], Chunning Li[1], Kaoru Nakamura[1], Cuiming Sun[1], Masaki Kuwabara[1], Masahiro Kitabatake[2], Teizo Yoshimura[1] & Akihiro Matsukawa[1]

1 Department of Pathology and Experimental Medicine, Graduated School of Medicine, Dentistry and Pharmaceutical Sciences, Okayama University, 2-5-1 Shikata, Kita-ku, Okayama, 700-8558, Japan.
2 Department of Immunology, Nara Medical University, Kashihara, 634-8521, Japan.

Figure 6a

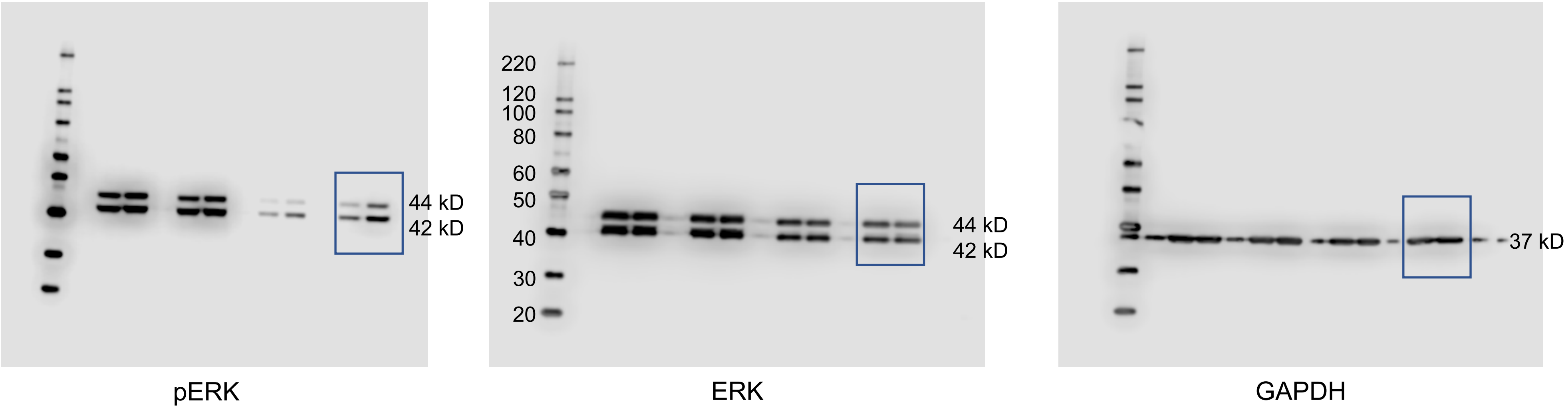

Figure 6b

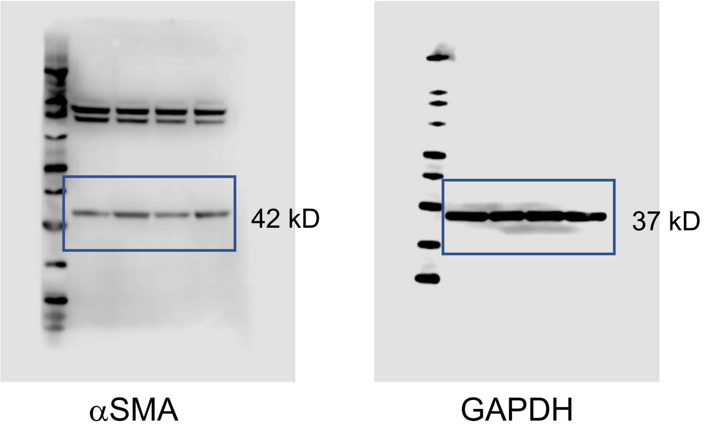

The MagicMark XP (Thermofisher) was used as a M.W. marker.
